# Evidence on the Use of Mobile Apps During the Treatment of Breast Cancer: Systematic Review

**DOI:** 10.2196/13245

**Published:** 2019-08-27

**Authors:** Flávia Oliveira Almeida Marques Cruz, Ricardo Alencar Vilela, Elaine Barros Ferreira, Nilce Santos Melo, Paula Elaine Diniz Dos Reis

**Affiliations:** 1 School of Health Sciences University of Brasilia Brasilia Brazil; 2 Centro Universitário do Distrito Federal Brasilia Brazil; 3 Hospital Universitário de Brasília Brasilia Brazil; 4 Grupo CONFIAR Goiânia Brazil

**Keywords:** mobile applications, health education, nursing care, review, educational technology, breast neoplasms

## Abstract

**Background:**

Cancer is a major cause of morbidity, disability, and mortality worldwide, and breast cancer is the most common cause of death in women. Different modalities of cancer treatment can have adverse effects that reduce the quality of life of patients and lead to treatment interruptions, if not managed properly. The use of mobile technologies has brought innovative possibilities for improving health care. Mobile apps can help individuals manage their own health and well-being and may also promote healthy lifestyles and information access.

**Objective:**

The aim of this study was to identify available evidence on the use of mobile apps to provide information and facilitate communication regarding self-care management related to the adverse effects of toxicities owing to breast cancer therapy.

**Methods:**

This systematic review includes studies which were identified using a search strategy adapted for each electronic database: CINAHL, Cochrane Library, LILACS, LIVIVO, PubMed, SCOPUS, and Web of Science. In addition, a gray literature search was performed using Google Scholar. All the electronic database searches were conducted on April 17, 2019. Two investigators independently reviewed the titles and abstracts of the studies identified and then read the full text of all selected papers. The quality of the included studies was analyzed by the Cochrane Collaboration Risk of Bias Tool and the Methodological Index for Non-Randomized Studies.

**Results:**

A total of 9 studies which met the eligibility criteria—3 randomized clinical trials and 6 nonrandomized studies published in English from 2010 to 2018—were considered for this systematic review; 396 patients with breast cancer, as well as 40 experts in the medical and nursing fields, and 3 software engineers were included.

**Conclusions:**

The evidence from the studies included in this systematic review is currently limited but suggests that mobile apps for women with breast cancer might be an acceptable information source that can improve patient well-being; they can also be used to report symptoms and adverse treatment-related effects and promote self-care. There is a need to test more evidence-based apps in future randomized clinical trials.

## Introduction

### Background

Cancer is a major cause of morbidity, disability, and mortality worldwide, affecting more than 18 million people each year [1]. In Brazil alone, about 600,000 new cases of the disease are estimated for 2019. Among women, the most common types of cancer are those of the breast, intestines, cervix, lungs, and thyroid. Worldwide, breast cancer is the most common cause of death in women, with approximately 626,000 estimated victims in 2018 [1,2].

The different modalities of cancer treatment can have adverse effects that may reduce the quality of life of patients and lead to treatment interruptions if not managed properly [3]. Considering the need to handle side effects in a population with a life-threatening disease, promoting symptom management–related knowledge remains a high priority for patients and a challenge for health professionals [4-6].

The landscape of mobile app usage has been growing and evolving. Apps can be used to offer services related to entertainment, media, education, shopping, finances, travel, health, and so on [7]. The use of mobile technologies presents innovative possibilities for improving health care. Mobile apps can help individuals manage their own health and well-being and may also promote healthy lifestyles and information access. According to international estimates, by 2018, nearly 2 billion smartphone and tablet users were using health-related apps [8].

Considering the important role they can play in patient education, disease self-management, and remote monitoring of patients, the use of smartphones is receiving more attention in the health domain every day. A systematic review investigating smartphone-based health care technologies concluded that many medical apps have been developed and are widely used by health professionals and patients alike [9].

In a study evaluating 185 mobile apps related to breast disease in the main app stores (Apple iTunes, Google Play, BlackBerry World, and Windows Phone), the authors found that most (n=139) concerned breast cancer [10]. A recent cross-sectional review of 599 apps [11] has verified the state of the practice regarding breast cancer-related mobile apps to characterize health apps from app stores (iOS and Android). These studies have identified a lack of evidence related to the involvement of medical experts in the creation and development of such apps. They have, therefore, highlighted the need to identify high-quality apps to increase consumer confidence in their use during health care [10,11].

A systematic review of apps targeting patients with breast and prostate cancer involved 5 studies and a total of 644 patients. The purposes of the apps were related to the main psychological variables in psycho-oncological care: quality of life and anxiety and depression symptoms [12]. Another systematic review identified 29 studies on mobile health apps targeting only patients with breast cancer. More than half of the studies addressed apps in an intervention for prevention, early detection of breast cancer, or survivors of the disease [13]. Both systematic reviews found that rigorous trials regarding the subject are lacking, despite the existence of studies related to cancer-focused apps. Future investigations should continue to explore and test the impact of mobile health apps on the treatment of breast cancer [12,13].

None of these recent reviews exclusively evaluated apps for women with breast cancer during cancer treatment. Therefore, given the magnitude of the disease burden, the needs of this population, the increasing use of mobile apps in the health domain, and the need to identify quality apps, it is necessary to enhance knowledge about the mobile apps available to provide information and improve the course of treatment of women with breast cancer.

### Objective

This systematic review aimed to identify available evidence on the use of mobile apps to provide information and facilitate communication regarding self-care management related to the adverse effects of toxicities owing to breast cancer therapy.

## Methods

### Protocol and Registration

This systematic review was conducted according to the Preferred Reporting Items for Systematic Reviews and Meta-Analyses Checklist [14]. The protocol was registered at the International Prospective Register of Systematic Reviews under number CRD42018083548 [15].

### Eligibility Criteria

In this systematic review, we included the following: (1) studies about mobile apps, defined as any computer programs or software installed on mobile electronic devices to provide information and facilitate communication regarding self-care management and adverse effects related to toxicities owing to breast cancer therapy, (2) studies that performed validation of content or evaluated the usability or effectiveness of apps, and (3) studies that collected the opinions of patients, clinicians, or experts about apps developed for patients with breast cancer. There were no restrictions on the year of publication or language.

Studies were excluded for the following reasons: (1) if they focused on mobile apps related to other types of cancer, (2) if they focused on electronic technologies, but not mobile apps, such as telephone services, text messages, videotapes, audiotapes, audiovisual materials in DVDs, websites, games, or online programs for desktop computers, (3) if they concerned the post-treatment period, (4) if their objective was to evaluate mobile apps intended for health professionals but not patients, (5) if they focused on emotional, cognitive, and behavioral strategies, and (6) if they took the form of reviews, letters, conference summaries, book chapters, or studies that only described the development of mobile apps.

### Information Sources and Search Strategy

Studies were identified using an individual search strategy for each of the following electronic databases: CINAHL, Cochrane Library, LILACS, LIVIVO, PubMed, SCOPUS, and Web of Science (Multimedia Appendix 1). The reference lists of selected papers were hand searched for potentially relevant studies that might have been missed in the electronic database searches. In addition, a gray literature search was performed using Google Scholar.

Duplicated references were removed by using appropriate software (EndNote Basic, Thomson Reuters). All the electronic database searches were conducted on April 17, 2019.

### Study Selection

Study selection was completed in 2 phases by using an online app (Rayyan, Qatar Computing Research Institute). In phase 1, 2 investigators (FOAMC and PEDR) independently screened the titles and abstracts of all citations retrieved from electronic databases and identified papers that appeared to meet the inclusion criteria. In phase 2, the same investigators independently read the full text of all selected papers and excluded studies that did not meet the inclusion criteria. Any disagreements in the first or second phases were resolved by discussion and consensus between the 2 reviewers. In case a consensus could not be reached, a third investigator (EBF) became involved to make a final decision. Studies that were excluded after full-text assessment and the reasons for their exclusion are listed in Multimedia Appendix 2.

### Data Collection Process and Items

Two investigators (FOAMC and PEDR) independently collected data from the selected papers: population characteristics (groups, n, mean age, and treatment focus on app), study characteristics (author(s), country and year of publication, and objective), intervention characteristics (purpose of the app, operation, description, and operating system), and outcome characteristics (primary outcomes and main conclusions). Any disagreement was resolved by discussion and mutual agreement. A third author (EBF) was involved when required to make a final decision. If the required data were not complete, attempts were made to contact the authors to retrieve any pertinent information.

### Risk of Bias in Individual Studies

Two investigators (FOAMC and PEDR) independently conducted the risk of bias assessment for the selected papers. Again, any disagreement was resolved by discussion and mutual agreement. A third author (EBF) was involved when required to make a final decision.

To assess the risk of bias of the included randomized controlled trials, including judgments about sequence generation, allocation concealment, blinding of participants, personnel and outcome assessors, incomplete outcome data, and selective reporting, the Cochrane Collaboration Risk of Bias Tool [16] was used. The risk of bias was assessed as low, high, or unclear. We also used the Methodological Index for Non-Randomized Studies (MINORS) [17] for the nonrandomized studies; this was done to analyze the study aim and appropriate endpoints, inclusion of participants, data collection, and follow-up period, as well as the calculation of the study size and loss to follow-up. For the comparative study, the characteristics of the groups and the statistical analyses were also verified.

### Synthesis of Results

The heterogeneity across studies was evaluated by considering clinical (treatment-related differences), methodological (design and risk of bias), and statistical (outcome measures) characteristics. Therefore, owing to the heterogeneity among the included studies, a quantitative synthesis was not undertaken. Congruent with the review objectives, the results of the included studies were analyzed and reported according to the characteristics of the mobile apps, their assessment, and satisfaction with their use.

## Results

### Study Selection

**Figure 1:**
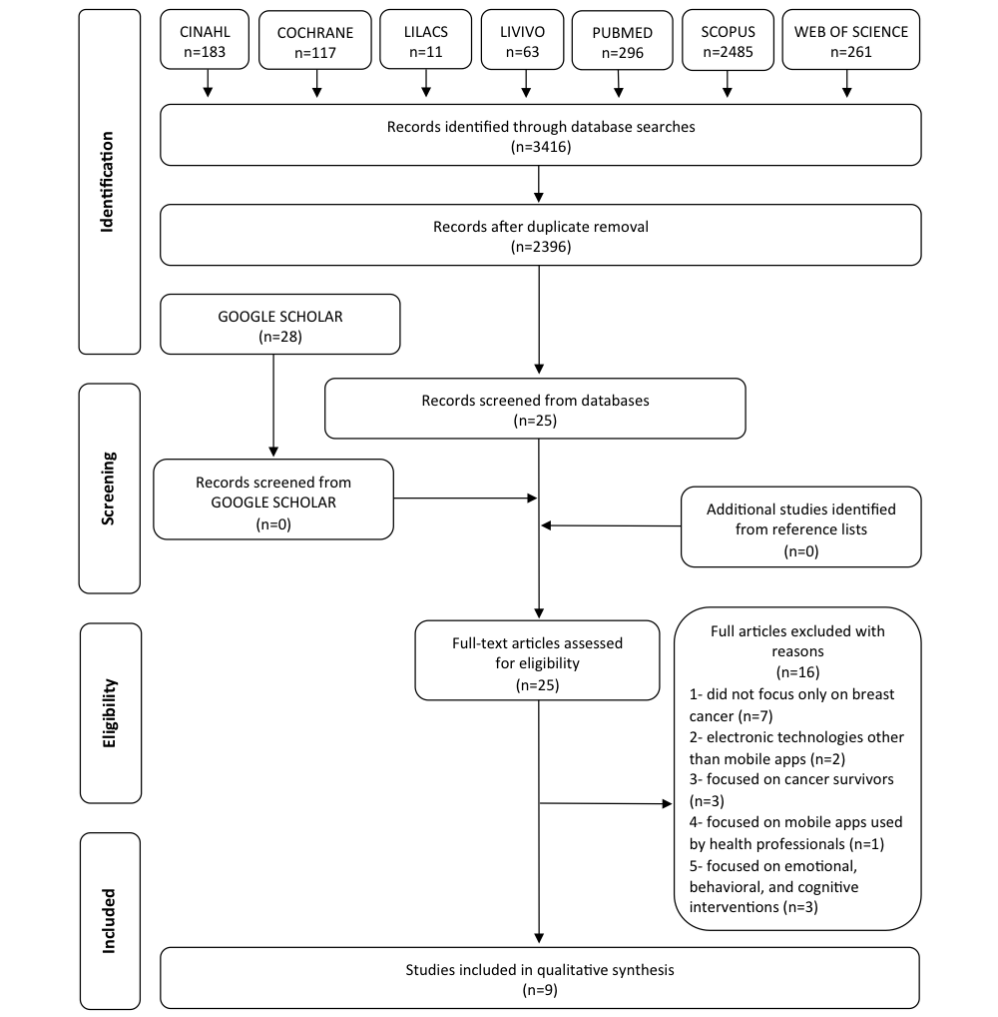
Flow diagram of literature search and selection process (adapted from Preferred Reporting Items for Systematic Reviews and Meta-Analyses [PRISMA]).

The literature search initially yielded 3416 papers from 7 electronic databases. After duplicate removal, the titles and abstracts of 2396 papers were screened, and 25 potentially relevant studies were selected for full-text reading; 16 papers were excluded (Multimedia Appendix 2) and 9 papers met all the eligibility criteria and were considered for this systematic review [18-26]. Figure 1 shows a flow diagram of the study identification, screening, and inclusion processes.

### Study Characteristics

All studies were published in English from 2010 to 2018 and evaluated mobile apps for women with breast cancer during treatment. In total, 4 studies included patients undergoing chemotherapy [18-21], 1 concerned postoperative patients [22], and another focused on both chemotherapy and surgery [23]. One study included patients undergoing chemotherapy and radiation therapy [24], and another included patients undergoing adjuvant endocrine therapy with aromatase inhibitors [25]. Only one study allowed any type of therapy for breast cancer [26].

In this systematic review, 396 patients with breast cancer were included, as well as 40 medical and nursing experts and 3 software engineers. The clinical nursing experts’ average career length was 17.0 years, with the duration ranging from 7 to 36 years in one of the studies [23]. One study included 2 breast surgeons, an oncologist, a radiation oncologist, a plastic surgeon, a gynecologist, a clinical geneticist, and 3 specialized breast cancer nurses [26].

A study measured health literacy through a validated instrument, showing that 72.5% of participants had high health literacy [25]. Regarding educational level, in 1 study, 56.1% of the participants had completed elementary or junior middle school [21]. Concerning familiarity with mobile apps, 1 study found that 67% of the participants frequently used them, while 33% were relatively inexperienced before participating in the study [26].

The main characteristics of the studies included are presented in Tables 1, 2, and 3.

**Table 1:** Summary of population characteristics of included papers (n=9).

Year, author, country^a^	Groups	N	Mean age (years)	App’s treatment focus
2016, Egbring et al, Switzerland [18]	CG^b^: regular physician support; EG1^c^: mobile app without physician review; EG2: mobile app and physician review on scheduled visits	CG: 41 patients; EG1: 45 patients; EG2: 41 patients	CG: 56; EG1: 50; EG2: 53	Chemotherapy
2018, Graetz et al, United States [25]	CG: mobile app without weekly reminders to use it; EG: mobile app with weekly reminders to use it	CG: 23 patients; EG: 21 patients	CG: 59.3; EG: 60.6	Adjuvant endocrine therapy with aromatase inhibitors
2016, Hwang, Canada [22]	CG: conventional follow-up; EG: e-monitoring in addition to conventional follow-up	CG: 37 patients; EG: 35 patients	CG: 65.5; EG: 60.1	Surgery
2010, Klasnja et al, United States [24]	Two groups of patients evaluated the app for three weeks	5 patients	50	Chemotherapy (n=3) and radiation therapy (n=2)
2017, Liu et al, China [23]	EvG1^d^: clinical nursing experts; EvG2: medical and nursing experts and software engineers	EvG1: 19 nurses; EvG2: 8 experts	EvG1: 38.9; EvG2: not available	Surgery and chemotherapy
2016, Young-Afat et al, The Netherlands [26]	EvG1: patients with breast cancer; EvG2: physicians and specialized nurses	EvG1: 15 patients; EvG2: 10 experts	EvG1: 51; EvG2: not available	Any type of cancer therapy
2017, Zhu et al, China [19]	EvG1: specialized nurses and an oncologist; EvG2: patients with breast cancer	EvG1: 6 experts; EvG2: 6 patients	EvG1: not available; EvG2: above 50	Chemotherapy
2018, Zhu et al, China [20]	Two groups (FTFI^e^ and TI^f^) of patients that evaluated the app	13 patients	49.5	Chemotherapy
2018, Zhu et al, China [21]	CG: only usual care; EG: e-support program and usual care	A: 57 patients; B: 57 patients	A: 46.2; B: 48.2	Chemotherapy

^a^Country of the study coordinator.

^b^CG: control group.

^c^EG: experimental group.

^d^EvG: evaluator group.

^e^FTFI: face-to-face interviews.

^f^TI: telephonic interviews.

**Table 2:** Summaries and intervention characteristics of included papers (n=9).

Year, author, country^a^	Study characteristics	Intervention characteristics	
	Objective	Purpose of the app	Operation
2016, Egbring et al, Switzerland [18]	To evaluate the effects of a mobile app on patient-reported daily functional activity	Improvement in the patient-reported functional activity and adverse effects of chemotherapy	The app allows patients to record their daily functional activity and perceived symptoms during chemotherapy with indications of severity. Patients can edit a list of their preselected symptoms or select any of the 48 symptoms available.
2018, Graetz et al, United States [25]	To evaluate the feasibility of a Web-based symptom-reporting app for patients with early-stage breast cancer using AIs^b^	Improvement in symptom burden and medication adherence	The ability to report symptoms and AI medication use, with built-in alerts sent to a patient’s care team on the basis of the predetermined thresholds.
2016, Hwang, Canada [22]	To determine if unscheduled visits for care and hospital readmission can be prevented by e-monitoring and to assess patient satisfaction with the app	Provision of care for postoperative wounds	The app allows for electronic wound monitoring. The patient takes photos of the wound on postoperative days 1, 3, 7, and 14 and attaches them to electronic messages sent to the surgeon, who must answer within 24 hours.
2010, Klasnja et al, United States [24]	To refine the functional requirements of a mobile app to assist patients with cancer during treatment	Provision of health information to manage care-related issues in unanchored settings	The app has modules including daily check-ins to track well-being and symptoms; calendar events (eg, consultations with clinicians); logs to monitor medications, pain, and surgery drains; and notes (ie, text, photo, and audio) for quick capture of care-related information.
2017, Liu et al, China [23]	To develop and evaluate the structure and contents of a smartphone app for women with breast cancer	Provision of information support regarding disease, treatment, medication, exercise, nutrition, symptoms, examination, and social support	The app has 5 main function modules: personalized information recommendation, category knowledge center, headline information browsing, newest information browsing, and information searching.
2016, Young-Afat et al, The Netherlands [26]	To evaluate patient experience and satisfaction, physicians’ and nurses’ opinions, and scientific potential of a supportive breast cancer app	To be beneficial in clinical practice and research	The app has 4 main functionalities: repository for information (audio recorded and imaging), symptom registration, timeline of treatment trajectory, and personalized information about breast cancer and treatment.
2017, Zhu et al, China [19]	To develop and evaluate the content and functionality of a mobile app for women with breast cancer undergoing chemotherapy	Provision of social, emotional, and information support	The app has 4 components: learning (information related to breast cancer and symptom management), discussion (anonymous support group), ask the expert (online consultation), and personal stories (stories of breast cancer survivors).
2018 A, Zhu et al, China [20]	To explore participants’ perceptions of the strengths and weaknesses of the BCS^c^, and their suggestions for program improvement	Provision of social, emotional, and information support	The app has 4 components: learning (information related to breast cancer and symptom management), discussion (anonymous support group), ask the expert (online consultation), and personal stories (stories of breast cancer survivors).
2018 B, Zhu et al, China [21]	To determine the effectiveness of the BCS program to address women’s self efficacy, symptoms, and quality of life during chemotherapy	Provision of social, emotional, and information support	The app has 4 components: learning (information related to breast cancer and symptom management), discussion (anonymous support group), ask-the expert (online consultation), and personal stories (stories of breast cancer survivors).

^a^Country of the study coordinator.

^b^AIs: aromatase inhibitors.

^c^BCS: Breast Cancer e-Support Program.

**Table 3:** Summary of interventions and outcome characteristics of included papers (n=9).

Year, author, country^a^	Intervention characteristics		Outcome characteristics		
	Description (developer)	Operating system	Primary outcomes	Main conclusions	
2016, Egbring et al, Switzerland [18]	Mobile app to record daily functional activity and adverse effects of chemotherapy	iOS^b^ and Android	Functional activity and adverse effects of chemotherapy	Patient well-being and reporting of the adverse effects of chemotherapy can be improved by using a mobile app under the supervision of the treating physician.	
2018, Graetz et al, United States [25]	App that allows patients to share information in real time with their cancer care team outside of clinic visits	Information not available	Symptom burden and medication adherence	The use of an app with weekly reminders significantly improved short-term AI^c^ adherence, which may reduce the symptom burden of women with breast cancer.	
2016, Hwang, Canada [22]	Smartphone app that allows for communication between the patient and the surgeon (Medeo)	Information not available	Unscheduled visits for care, hospital readmission, and patient satisfaction	Electronic wound monitoring was associated with significantly less unscheduled care, including hospital readmission and visits to the emergency department or walk-in clinic, a high degree of patient satisfaction, and a possible reduction in cost to the health care system.	
2010, Klasnja et al, United States [24]	Mobile app to assist patients in managing care-related information (HealthWeaver Mobile)	Android only	Participants’ perceptions of HealthWeaver Mobile	The possibility of taking photos and audio notes was highly valued by participants. The app was seen not only as a valuable way to capture information quickly but also as a means of accessing information, especially through calendar events.	
2017, Liu et al, China [23]	Smartphone app to provide personalized information support (Information Assistant)	Information not available	Participants’ evaluation of Information Assistant	A few useful pieces of information, photos, and videos have been added, allowing patients to gain maximum benefits from the app. It is able to deliver high-quality information support.	
2016, Young-Afat et al, The Netherlands [26]	Supportive breast cancer mobile app to assist in clinical practice and research (OWise)	iOS and Android	Participants’ evaluation of OWise	Benefits for patients and their medical teams, especially because of the option to make audio recordings of consultations and the availability of personalized information.	
2017, Zhu et al, China [19]	Mobile app to promote women’s self-efficacy and social support (BCS^d^)	iOS and Android	Participants’ evaluation of BCS	More information has been added in the app tutorial, as well as the information related to food choices, sexual activity, pregnancy, and the interpretation of laboratory results, making the app useful, attractive, and easy to use.	
2018, Zhu et al, China [20]	Mobile app to promote women’s self-efficacy and social support (BCS)	iOS and Android	Participants’ perceptions of BCS	Potential of BCS to support women during chemotherapy. Its benefits can be maximized by incorporation into routine care.	
2018, Zhu et al, China [21]	Mobile app to promote women’s self-efficacy and social support (BCS)	iOS and Android	Self-efficacy	The BCS program demonstrated its potential as an effective and easily accessible intervention to promote women’s self-efficacy, symptom interference, and quality of life during chemotherapy.	

^a^Country of the study coordinator.

^b^iOS: iPhone Operating System.

^c^AIs: Aromatase Inhibitors.

^d^BCS: Breast Cancer e-Support Program.

### Risk of Bias Within Studies

On the basis of the methodological quality assessment using MINORS [17], the total scores of the validation studies ranged from 12 to 16 [19,20,23,24,26], while the nonrandomized comparative study reached a total score of 21 [22], as shown in Table 4.

**Figure 2:**
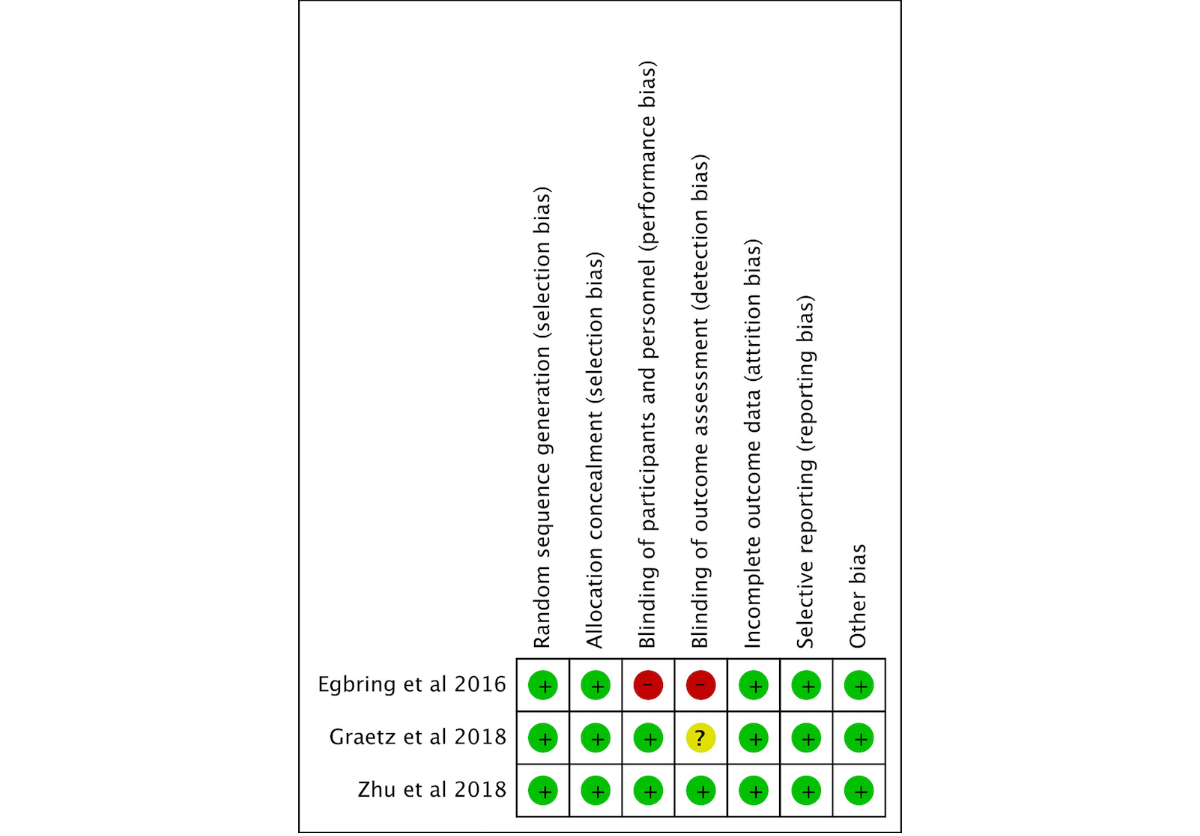
Methodological appraisal of selected studies on the basis of Cochrane Collaboration Risk of Bias Tool.

On the basis of the methodological quality assessment using the Cochrane Collaboration Risk of Bias Tool [16], one of the studies [21] presented a low risk of bias in all domains evaluated. Only one of the studies [18] presented high risk in 2 domains, both regarding blinding; the authors indicated that the absence of blinding may have affected the outcomes evaluated. In one study, there were no reports regarding blinding of outcome assessment [26], as shown in Figure 2.

**Table 4:** Methodological appraisal of selected studies on the basis of Methodological Index for Nonrandomized Studies (MINORS).

Criteria^a^	Hwang, 2016 [22]	Klasnja et al 2010 [24]	Liu et al 2017 [23]	Young-Afat et al 2016 [26]	Zhu et al 2017 [19]	Zhu et al 2018 [20]
1. A clearly stated aim	2	2	2	2	2	2
2. Inclusion of consecutive patients	2	2	2	2	2	0
3. Prospective collection of data	2	2	2	2	2	2
4. Endpoints appropriate to aim of study	2	2	2	2	2	2
5. Unbiased assessment of study endpoint	2	2	2	2	2	2
6. Follow-up period appropriate for aim of study	2	2	2	2	0	2
7. Loss to follow-up less than 5%	2	0	2	0	2	2
8. Prospective calculation of study size	1	0	2	2	2	2
9. An adequate control group	2	N/A^b^	N/A	N/A	N/A	N/A
10. Contemporary groups	1	N/A	N/A	N/A	N/A	N/A
11. Baseline equivalence of groups	1	N/A	N/A	N/A	N/A	N/A
12. Adequate statistical analyses	2	N/A	N/A	N/A	N/A	N/A
Total score	21	12	16	14	14	14

^a^Items were scored 0 (not reported), 1 (reported but inadequate), or 2 (reported and adequate). The global ideal total score is 16 for noncomparative studies and 24 for comparative studies. See list of references for full source information on papers. MINORS index is described in Slim et al [14].

^a^Not applicable.

### Synthesis of Results

All the analyzed studies carried out the evaluation of mobile apps for women during the treatment of breast cancer. On the basis of the objective, different types of apps were developed. In general, all involved the provision of useful and quality information to patients as a way of improving management of adverse treatment effects through the promotion of self-care at home.

Some studies reported the importance of using the specific app under the supervision of health professionals, either online [19,22,25] or in person [18]. Two studies evaluated an app capable of communicating with health care professionals, providing remote electronic monitoring [22,25]. One study developed a pilot randomized controlled trial to assess the feasibility of an app with or without weekly reminders, allowing the sharing of health information on a real-time basis with the patient’s oncology care team. Participants who used the weekly reminders feature had a higher app usage rate (74% vs 38%, *P*<.05) during the intervention and reported higher drug adherence than those who did not opt for this feature (100% vs 72%, *P*<.05) [25].

Another study reported a significantly lower number of unexpected medical consultations, including hospital readmissions and visits to the emergency department, in the e-monitoring group than the conventional follow-up control group (3% vs 22%, *P*<.05), as the app allows the professional to conduct an electronic consultation if necessary, avoiding complications. Almost all e-monitoring patients felt that the app led to improved care (95%) and would recommend it to a friend or colleague (90%).

Three studies in this systematic review [19-21] concerned an app with an *ask the expert* module, capable of providing online consultations, which was found to be useful, attractive, and easy to use [19]. The authors developed and evaluated the content and functionality of the mobile app [19], tested it to determine its effectiveness regarding women’s self efficacy, symptoms, and quality of life during chemotherapy [21], and explored participants’ perceptions of app strengths and weaknesses [20].

Through qualitative interviews, it emerged that the participants considered the app to be useful for improving knowledge and promoting emotional well-being and would recommend it to other women undergoing chemotherapy [20]. In addition, when tested, the app had significantly better health outcomes at 3 months regarding self-efficacy (21.05; 95% CI 1.87 to 40.22; *P*=.03; *d*=0.53), symptom interference (−0.73; 95% CI −1.35 to −0.11; *P*=.02; *d*=−0.51), and quality of life (6.64; 95% CI 0.77 to 12.50; *P*=.03, *d*=0.46) compared with participants who received usual care [21].

Another study reported the importance of an app for patients who require face-to-face follow-up with health care professionals on scheduled visits. Through the app, the patient can make a recording of the perceived symptoms at home, improving the report of the adverse effects management for the health care professional during consultation [18].

The focus of some studies was on using mobile apps to improve the retention of information about the management of adverse effects and, consequently, to improve self-care and patient well-being. One study evaluated an app with a module dedicated to the latest research findings, technologies, and methods for breast cancer care, thereby helping patients obtain up-to-date information [23].

Moreover, one study assessed a mobile app that, in addition to offering patient guidance through the provision of knowledge, includes a calendar to highlight important dates related to events, treatments, and consultations. This app incorporates texts, photos, and audio, making it possible for patients to capture and store important information related to their care. This app allows greater interaction between the patient and the app, a fact that was valued by the study participants [24].

**Figure 3:**
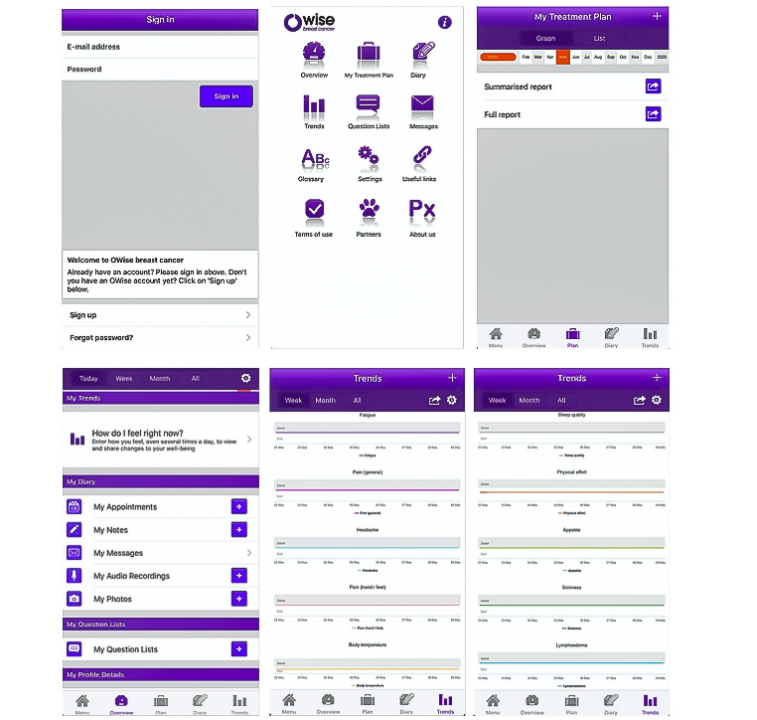
Screenshots of the OWise.

However, this was not the only study on an app enabling the inclusion of audio. In another study, all patients (n=15) used the audio recording function to record consultations with their nurses and physicians, and 14 (93%) patients found this feature useful. Patients considered the audio aspect and the personalization of information about disease and treatment as the most useful features of the app. Almost all physicians and nurses (90%) also found the recording function useful and would recommend the app to their patients [26]. Figure 3 shows screenshots of this smartphone app, named OWise.

## Discussion

### Summary of Evidence

This is a systematic review about the available evidence regarding women’s use of mobile apps during breast cancer treatment. A total of 9 papers, which covered the development of various types of mobile apps, were included [18-26]. The apps encompassed the transmission of useful information and the management of the adverse effects of breast cancer treatment through various features.

While there is growing interest in using patient reporting tools to improve symptom monitoring during cancer treatment, there is a lack of evidence about the impact of this strategy on clinical outcomes [26,27]. A mobile app has the potential to restore the daily functional activity of patients with early-stage breast cancer. This benefit is most significant when the patient uses the app under the supervision of health professionals as the review of the treatment during consultation is an opportune moment for a sincere discussion of symptoms reported by the patients in the app [18].

Electronic follow-up via apps may help avoid unplanned visits to the emergency room and hospital readmissions, reducing health care costs [22]. These results corroborate those of a study that examined health-related quality of life, emergency room visits, and hospitalizations among patients receiving chemotherapy for advanced solid tumors. The use of tablet computers to self-report symptoms during cancer treatment was associated with significant clinical benefits, decreased emergency room admissions, and reduced hospitalizations [27].

Web-based self-management support systems and apps involving health professionals are important to ensure the communication of high-quality information between patients and the health team empowering the patients to increase their self-care and improve their own health [28] and also the communication between patients and staff outside of health institutions as a way of ensuring continuity of care [22], enhancing knowledge, improving confidence level, and promoting emotional well-being [20]. Besides that, the involvement of health professionals in monitoring treatment effects through an app outside of clinic visits also might be a cost-effective way to improve symptom management and health outcomes [25].

Calendar events, photos, messages, and audio are a few of the mechanisms that can increase the usability of apps and improve the acquisition of information related to illness and treatment [18,24,26]. These features may improve self-care at home and restore the daily functional activity of patients. In addition, the audio recording function to record consultations with health professionals does not increase the duration of the consultation [26].

Characteristics related to communication and interaction were also demonstrated by a recent study that aimed to investigate the behavior of patients with prostate cancer when using a mobile app during radiation therapy [29]. As found in the studies included in this systematic review, there was improvement in the reporting and self-management of symptoms during treatment. In addition, the app provided continuous access to links to important information and, like one of the apps evaluated by a study included in this review, helped patients obtain up-to-date information regarding their disease and treatment [23].

In this context, health apps are increasingly being used in clinical care and might have significant informative potential. However, they are often inserted into clinical care before the necessary research to confirm the benefits for patients and health professionals has been conducted [26].

### Limitations

Some methodological limitations of this systematic review should be taken into account. Most studies validated apps for mobile devices during their development, focusing on their content and functionality. More data from randomized clinical trials are needed to assess the effects of using mobile apps during the treatment of women with breast cancer, as is being done with the Breast Cancer e-Support program. In addition, the evaluated apps have different functionalities and are geared toward different treatments. Therefore, their heterogeneity prevents the data from being satisfactorily grouped, hindering a quantitative analysis.

### Conclusions

The evidence from the studies included in this systematic review is currently limited but suggests that mobile apps for women with breast cancer might be an acceptable information source and lead to improved patient well-being. They can also be used to report symptoms and adverse treatment-related effects and promote self-care. However, the real utility of mobile apps for women undergoing breast cancer treatment is still uncertain. More evidence-based apps must be tested in future randomized clinical trials.

## Appendix

Multimedia Appendix 1 Database search strategies.

Multimedia Appendix 2 Full articles excluded (n=16) from review with reasons.

## Abbreviations

MINORS: Methodological Index for Non-Randomized Studies
