# Supplementary material for: Evidence on the Use of Mobile Apps During the Treatment of Breast Cancer: Systematic Review
Source: JMIR Mhealth Uhealth. 2019 Aug 27;7(8):e13245. doi: 10.2196/13245 (PMC6734853; doi:10.2196/13245)
Supplement: Multimedia Appendix 1 [file mhealth_v7i8e13245_app1.pdf]

Appendix 1 - Database search strategies.

Database	Search Terms
PubMed (April 17th, 2019)	("breast neoplasms"[MeSH Terms] OR "breast neoplasms"[All Fields] OR "breast tumor"[All Fields] OR "breast tumors"[All Fields] OR "breast cancer"[All Fields] OR "breast cancers"[All Fields] OR "mammary cancer"[All Fields] OR "mammary cancers"[All Fields] OR "breast malignant neoplasm"[All Fields] OR "breast malignant neoplasms"[All Fields] OR "breast malignant tumor"[All Fields] OR "breast malignant tumors"[All Fields]) AND ("mobile applications"[MeSH Terms] OR "mobile applications" [All Fields] OR "mobile application" [All Fields] OR "mobile app"[All Fields] OR "mobile apps"[All Fields] OR "portable electronic app"[All Fields] OR "portable electronic apps"[All Fields] OR "portable electronic application"[All Fields] OR "portable electronic applications"[All Fields] OR "portable software app"[All Fields] OR "portable software apps"[All Fields] OR "portable software application"[All Fields] OR "portable software applications"[All Fields] OR "smartphone"[MeSH Terms] OR "smartphone"[All Fields] OR "smartphones"[All Fields] OR "mobile health units"[MeSH Terms] OR "mobile health units" [All Fields] OR "mobile health unit" [All Fields] OR "mobile health" [All Fields] OR "eHealth"[All Fields] OR "mHealth"[All Fields] OR "health apps"[All Fields] OR "health app"[All Fields])
LILACS (April 17th, 2019)	(breast neoplasms OR neoplasias de la mama OR neoplasias da mama) AND (mobile applications OR aplicaciones móviles OR aplicativos móveis)
Cochrane Library CINAHL Scopus Web of Science (April 17th, 2019)	("breast neoplasms" OR "breast tumor" OR "breast tumors" OR "breast cancer" OR "breast cancers" OR "mammary cancer" OR "mammary cancers" OR "breast malignant neoplasm" OR "breast malignant neoplasms" OR "breast malignant tumor" OR "breast malignant tumors") AND ("mobile applications" OR "mobile application" OR "mobile app" OR "mobile apps" OR "portable electronic app" OR "portable electronic apps" OR "portable electronic application" OR "portable electronic applications" OR "portable software app" OR "portable software apps" OR "portable software application" OR "portable software applications" OR "smartphone" OR "smartphones" OR "mobile health units" OR "mobile health unit" OR "mobile health" OR "eHealth" OR "mHealth" OR "health apps" OR "health app")
Google Scholar LIVIVO (April 17th, 2019)	("breast neoplasms" "mobile applications")
