# Supplementary material for: Evidence on the Use of Mobile Apps During the Treatment of Breast Cancer: Systematic Review
Source: JMIR Mhealth Uhealth. 2019 Aug 27;7(8):e13245. doi: 10.2196/13245 (PMC6734853; doi:10.2196/13245)
Supplement: Multimedia Appendix 2 [file mhealth_v7i8e13245_app2.pdf]

Appendix 2 - Full articles excluded (n = 16) from review with reasons

Author, year	Full articles excluded with reasons
Abernethy et al, 2009	2
Absolom et al, 2017	1
Baseman et al, 2017	3
Cowie et al, 2013	1
Foley et al, 2016	5
Fromme, Heinige, Hribar, 2010	1
Kim et al, 2016	5
Kim et al, 2018	2
Maguire, 2005	1
McCann, et al, 2009	1
Min et al, 2014	5
Mirkovic, Kaufman, Ruland, 2014	1
Oldenmenger, Baan, Rijt, 2017	1
Park, 2019	3
Partl, 2017	4
Visser et al, 2018	3

(1) studies that focused on mobile apps related to other types of cancer;

(2) studies that focused on electronic technologies, but not mobile apps, such as telephone services, text messages, videotapes, audiotapes, audiovisual materials in DVDs, websites, games, or online programs for desktop computers;

(3) studies that concerned the post-treatment period;

(4) studies in which objective was to evaluate mobile apps intended for health professionals but not patients;

(5) studies that focused on emotional, cognitive, and behavioral strategies;

(6) reviews, letters, conference summaries, book chapters, or studies that only described the development of mobile apps.

References

Abernethy AP et al. Feasibility and acceptability to patients of a longitudinal system for evaluating cancer-related symptoms and quality of life: pilot study of an e/Tablet data-collection system in academic oncology. J Pain Symptom Manage. 2009 Jun;37(6):1027-38.

Absolom K et al. Electronic patient self-Reporting of Adverse-events: Patient Information and aDvice (eRAPID): a randomised controlled trial in systemic cancer treatment. BMC Cancer (2017) 17:318.

Baseman J et al. A mobile breast cancer survivorship care app: pilot study. JMIR Cancer 2017;3(2):e14).

Cowie J et al. Real-time management of chemotherapy toxicity using the Advanced Symptom Management System (ASyMS). Journal of Decision Systems, Vol. 22, No. 1, 43–52, 2013.

Foley NM et al. PATI: Patient accessed tailored information: A pilot study to evaluate the effect on preoperative breast cancer patients of information delivered via a mobile application. Breast. 2016 Dec;30:54-58.

Fromme EK, Heinige TK, Hribar M. Developing an easy-to-use tablet computer application for assessing patient-reported outcomes in patients with cancer. Support Care Cancer (2011) 19:815–822.

Kim J et al. Depression screening using daily mental-health ratings from a smartphone application for breast cancer patients. J Med Internet Res 2016 | vol. 18 | iss. 8 | e216.

Kim HJ et al. A mobile game for patients with breast cancer for chemotherapy self-management and quality-of-life improvement: randomized controlled trial. J Med Internet Res 2018 | vol. 20 | iss. 10 | e273.

Maguire R et al. Results of a UK based pilot study of a mobile phone based advanced symptom management system (ASyMS) in the remote monitoring of chemotherapy related toxicity. Clinical Effectiveness in Nursing (2005) 9, 202–210.

McCann L et al. Patients' perceptions and experiences of using a mobile phone-based advanced symptom management system (ASyMS©) to monitor and manage chemotherapy related toxicity. European Journal of Cancer Care, 2009, 18, 156–164.

Min YH et al. Daily collection of self-reporting sleep disturbance data via a smartphone app in breast cancer patients receiving chemotherapy: a feasibility study. J Med Internet Res 2014 | vol. 16 | iss. 5 | e135 | p.1.

Mirkovic J, Kaufman DR, Ruland CM. Supporting Cancer Patients in Illness Management: Usability Evaluation of a Mobile App. *JMIR* mHealth uHealth 2014;2(3):e33.

Oldenmenger WH, Baan MAG, Rijt CCDVD. Development and feasibility of a web application to monitor patients' cancer-related pain. Support Care Cancer. 2018 Feb;26(2):635-642.

Park S et al. Factors associated with physical activity of breast cancer patients participating in exercise intervention. Supportive Care in Cancer (2019) 27:1747–1754.

Partl R et al. 128 SHADES OF RED: Objective Remote Assessment of Radiation Dermatitis by Augmented Digital Skin Imaging. Health Informatics Meets eHealth. 2017.

Visser A et al. Group medical consultations (GMCs) and tablet-based online support group sessions in the follow-up of breast cancer: a multicenter randomized controlled trial. The Breast 40 (2018) 181e188.
